# [Corrigendum] VDAC upregulation and αTAT1-mediated α-tubulin acetylation contribute to tanespimycin-induced apoptosis in Calu-1 cells

**DOI:** 10.3892/or.2026.9161

**Published:** 2026-07-13

**Authors:** Qilin Wang, Xiangguo Liu

Oncol Rep 44: 2725–2734, 2020; DOI: 10.3892/or.2020.7789

Subsequently to the publication of the above paper, an interested reader drew to the authors' attention that the western blot Hsp90α bands in Fig. 1A were strikingly similar to data featured in two other papers written by the same research group; in Fig. 3A of reference 20 (by Qilin Wang *et al*, in the journal *Cancer Cell International*, published in 2012), and Fig. 8A in reference 43 (by Qilin Wang and Xiangguo Liu, also in the journal *Cancer Cell International*, published in 2020). The authors explained that they performed the Hsp90α knockdown experiments and western blot assays using duplicate membranes from the same experiments, although these were published separately in the three papers. The Hsp90α data were intentionally shared among the papers in order to show the same knockdown efficiency. Secondly, the microscopic image showing 1.0 µM Tanespimycin treatment in Fig. 2C of the abovementioned article had also apparently been included in Fig. 5B in a paper by Qilin Wang and Xiangguo Liu published in 2020, also in the journal *Cancer Cell International*. In that case, the authors re-examined their original data, and realized that the data in question in Fig. 2C had been inadvertently copied during editing.

The authors proposed providing alternative data for Fig. 1A from other independent Hsp90α and Hsp90β knockdown experiments, respectively, and the revised version of Fig. 1 is shown on the next page. The revised version of Fig. 2, now showing all the correct data in Fig. 2C, is shown on the third page. The authors regret the errors that were made during the compilation of the original figures, and are grateful to the editor of *Oncology Reports* for allowing them the opportunity to publish this Corrigendum. Note that these errors did not have a significant impact on the conclusions reached in this study. All the authors agree with the publication of this corrigendum; furthermore, they apologize to the readership for any inconvenience caused.

## Figures and Tables

**Figure 1.**
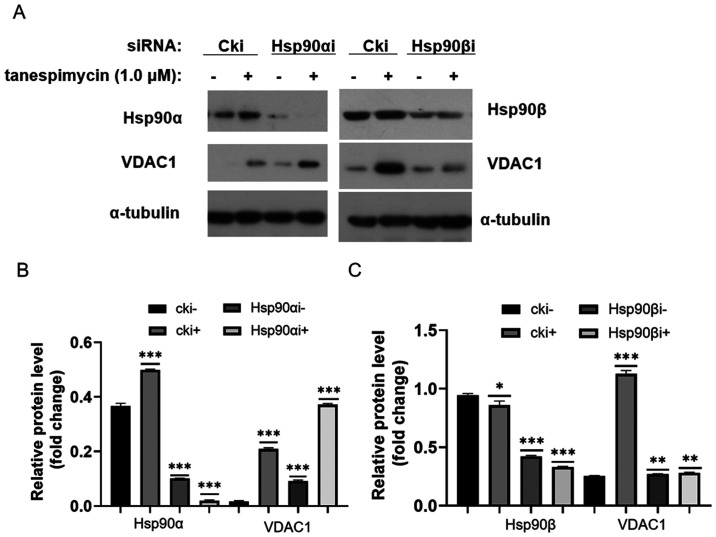
Hsp90α/β knockdown mediates VDAC1 expression. (A) Calu-1 cells were cultured in 6-well plates and on the 2nd day they were transfected with control siRNA (Cki) or Hsp90α/β siRNA (Hsp90αi/βi). At 24 h following transfection, cells were reseeded in a 6-well plate and treated with 1.0 µM tanespimycin for 48 h and the cells were then harvested for the preparation of whole-cell protein lysates for western blot analysis, and the indicated proteins Hsp90α/β VDAC1 and α-tubulin were analysed. α-tubulin was used as the loading control (Hsp90α, 90 kDa; Hsp90β, 90 kDa; VDAC1, 32 kDa; α-tubulin, 50 kDa). (B and C) The above western blot analysis results were analyzed by ImageJ software and GraphPad Prism 8 software, and the analysis results are the means of 3 independent assays. The western blot shown is representative of 3 independent experiments. Comparisons between groups were carried out with the Multiple t-test (Holm-Sidak correction was applied to the P-values of multiple comparison) and the comparison of interest is the relative protein in Cki(−) treatment. Values of P<0.05 were considered to indicate a statistically significant difference; *P<0.05, **P<0.005 and ***P<0.001 compared with the untreated cells. Error bars in the figures represent the SD values. Hsp, heat shock protein; VDAC1, voltage-dependent anion channel 1.

**Figure 2.**
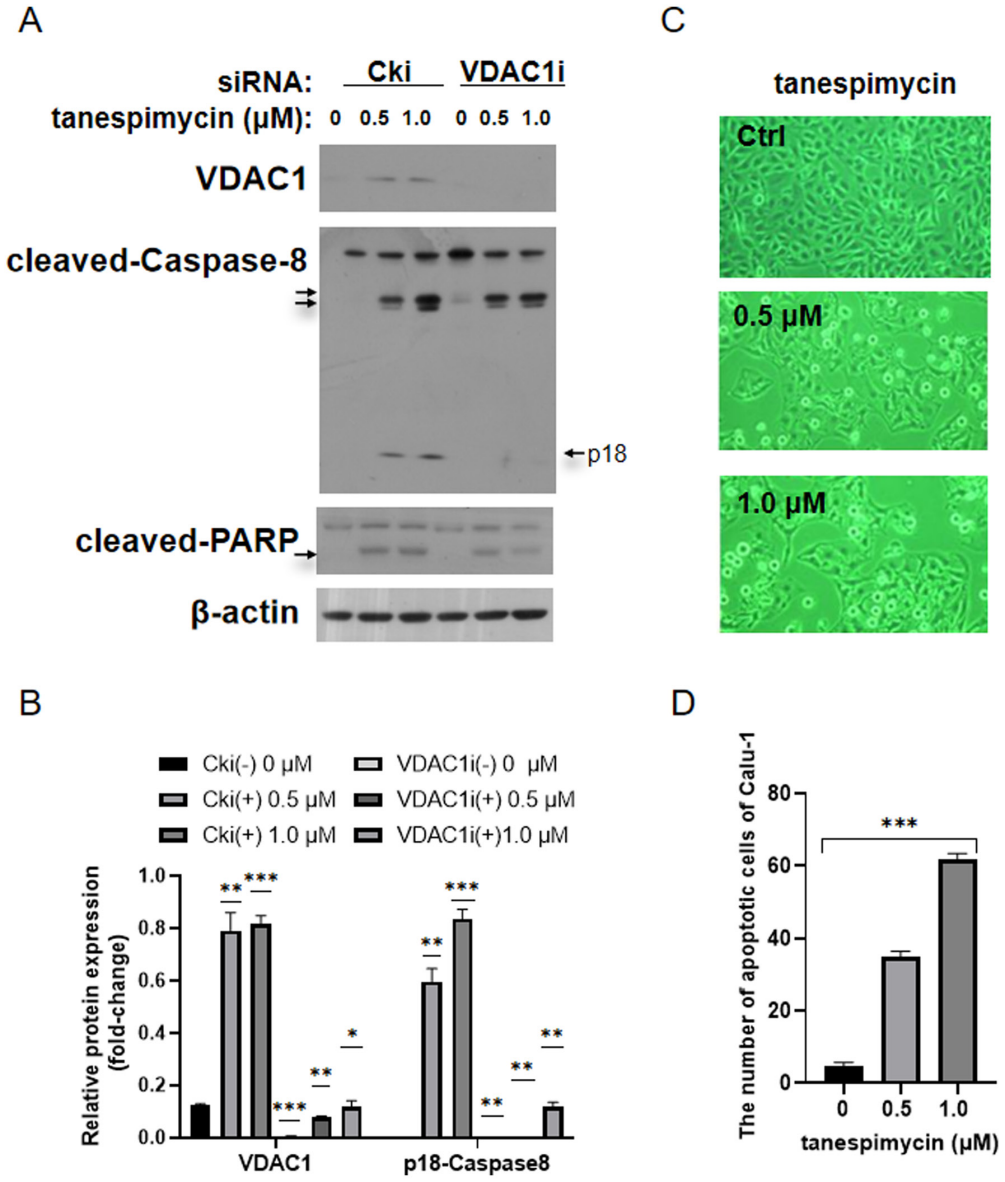
VDAC1 upregulation induced by tanespimycin is involved in cell apoptosis. (A) Calu-1 cells were cultured in 6-well plate and on the 2nd day they were transfected with control siRNA (Cki) or VDAC1 siRNA (VDAC1i). At 24 h following transfection, cells were reseeded in 6-well plate and treated with tanespimycin (0, 0.5 and 1.0 µM) for 48 h. The cells were then harvested for the preparation of whole-cell protein lysates for following western blot analysis to detect VDAC1, Caspase-8, PARP and β-actin levels. β-actin was used as the loading control (VDAC1, 32 kDa; caspase-8, 18, 43, 57 kDa; PARP, 89, 116 kDa; β-actin, 45 kDa). (B) The above western blot analysis results of VDAC1 and p18-caspase8 were analyzed by ImageJ software and GraphPad Prism 8 software and the analysis results were the mean of 3 independent experiments. The western blot shown is representative of 3 independent experiments. Comparison between groups were carried out with the Multiple t-test (Holm-Sidak correction was applied to the P-values of multiple comparison). The comparison of interest is the relative protein in Cki (−) treatment; *P<0.05, **P<0.005 and ***P<0.001. Error bars in the figures represent the SD values. (C) Calu-1 cells were treated with tanespimycin (0, 0.5 and 1.0 µM), and then the cells were subjected to take photos by inverted phase contrast microscope (×200 magnification) to observe the cell apoptotic state. (D) The number of Calu-1 apoptotic cells were calculated by ImageJ software. Comparison between groups was carried out with the RM one-way ANOVA method (a Geisser-Greenhouse correction was applied to the P-values). ***P<0.001. The analysis results are the means of 4 replicate determinations; bars represent SD. P<0.001. VDAC1, voltage-dependent anion channel 1.

